# Potential utility of a multi-component coagulation factor panel to calculate MELD scores and assess the risk of portal vein thrombosis in chronic liver disease

**DOI:** 10.1186/s12876-023-02695-6

**Published:** 2023-03-09

**Authors:** Clayton S. Lewis, Khurram Bari, Changchun Xie, Kenneth E. Sherman, Marc Vasse, Patrick Van Dreden, Vladimir Y. Bogdanov

**Affiliations:** 1 Division of Hematology/Oncology, Department of Internal Medicine, University of Cincinnati College of Medicine, 3125 Eden Ave., Rm 1316, Cincinnati, OH 45267 USA; 2 Division of Digestive Diseases, Department of Internal Medicine, University of Cincinnati College of Medicine, Cincinnati, OH USA; 3 Department of Environmental and Public Health Sciences, University of Cincinnati College of Medicine, Cincinnati, OH USA; 4 Department of Biology and UMR INSERM 1176, Foch Hospital, Suresnes, France; 5 Diagnostica Stago, Gennevilliers, France

**Keywords:** Liver diseases, Venous thrombosis, Factor V, Factor VIII, Protein C

## Abstract

**Background:**

Current quantitative approaches to assess chronic liver disease (CLD) severity have limitations. Further, portal vein thrombosis (PVT) pre-liver transplant (LT) is a major contributor to morbidity in CLD; the means of detecting and/or predicting PVT are limited. We sought to explore whether plasma coagulation factor activity levels can serve as a substitute for prothrombin time/international normalized ratio (PT/INR) in the Model for End-stage Liver Disease (MELD), and/or help assess the risk of PVT.

**Methods:**

Plasma activity levels of Factor V (FV), Factor VIII (FVIII), Protein C (PC), and Protein S (PS) and the concentrations of D-dimer, sP-selectin, and asTF were assessed in two cohorts of CLD patients (ambulatory, n = 42; LT, n = 43).

**Results:**

FV and PC activity levels strongly correlated with MELD scores, which enabled the development of a novel scoring system based on multiple linear regressions of the correlations of FV and PC activity with MELD-Na that substitutes PT/INR. Six-month and 1-year follow-up revealed that our novel approach was non-inferior to MELD-Na at predicting mortality. A significant inverse correlation between FVIII activity levels and PVT was found in the LT cohort (*p* = 0.010); FV and PS activity levels were in-trend (*p* = 0.069, *p* = 0.064). We developed a logistic regression-based compensation score to identify patients at risk of PVT.

**Conclusions:**

We demonstrate that FV and PC activity levels may be used to replace PT/INR in MELD scoring. We also show the potential of using the combination of FV, FVIII, and PS activity levels to assess the risk of PVT in CLD.

## Essentials

- Current quantitative approaches to assess chronic liver disease severity have limitations.
- 7 hematologic factors were measured in two cohorts of patients with chronic liver disease (n = 42, 43).
- Activity levels of Factor V and Protein C can be potentially used to substitute PT/INR in MELD scoring.
- Activity levels of Factor V, Factor VIII, and Protein S exhibit potential to help assess the risk of PVT.

## Background

Chronic liver disease (CLD) is the twelfth leading cause of death in the US [1]. Currently, optimal treatment for many end-stage CLD patients includes liver transplantation (LT). Portal vein thrombosis (PVT), which is present in between 4.5 and 25% of CLD patients and increases in prevalence with increased CLD severity [2–4], increases LT related morbidity and mortality [5]. This is due to the amount of non-anatomical vascular reconstruction necessary to ensure portal vein patency. Resultantly, overall survival of LT is decreased in patients with PVT [6], which is notoriously difficult to detect: up to 50% of PVT likely remains undetected in these patients due to imaging limitations [7].

PVT is not only difficult to detect, but also to predict; currently, no models exist that would chiefly rely on blood-derived parameters. Among the coagulation factors known to be altered in CLD is Factor V (FV), the cofactor component of the prothrombinase complex. FV activity is often suppressed in CLD, serving as a strong indicator of liver failure [8–10]. Conversely, the levels of Factor VIII (FVIII), the cofactor component of the intrinsic tenase complex, are drastically increased in CLD. This is not due to an increased synthesis of FVIII, but rather the increased endothelial production and release of von Willebrand Factor (vWF), which binds FVIII thereby increasing its half-life, and whose expression is correlated with portal hypertension and liver failure [11, 12]. Interestingly, in patients with severe CLD, FVIII activity levels were shown to be lower in patients with PVT compared to those without PVT [13]. Production of the anti-coagulant proteins protein C (PC) and protein S (PS) is dependent on vitamin K for the γ-carboxylation of multiple N-terminal glutamic acid residues, which is diminished in CLD; resultantly, the levels of PC and PS are reduced in CLD [14]. Furthermore, decreases in the circulating levels of PC and PS are associated with an increased incidence of PVT [15]. As CLD progresses, the ratio of FVIII to PC increases leading to the accumulation of intrahepatic microthrombi [16, 17]. There is a strong positive correlation between the extent of microthrombosis and the degree of liver fibrosis [16, 18, 19]. Elevated thrombogenesis leads to increased fibrinolysis in CLD; in a prospective study of patients with esophageal varices, patients with elevated hyperfibrinolysis as assessed by high levels of D-dimer and tissue plasminogen activator, had more frequent bleeding and more severe CLD. In this same study, D-dimer levels were found to positively correlate with the degree of CLD severity as assessed using the Child–Turcotte–Pugh (CTP) system [20]. This finding was later confirmed by Fimognari et al. who not only showed that D-dimer levels are significantly increased in CTP-C patients compared with CTP-A and CTP-B, but that D-dimer levels are even higher in CTP-C patients with PVT [13].

Progressive derangement of sinusoidal endothelium comprises another major hallmark of CLD. Liver injury promotes de-differentiation of sinusoidal endothelial cells to a more capillarized phenotype [21]. Resultantly, the sinusoidal endothelium begins to express P-selectin, which promotes monocyte infiltration [22]. Soluble (s)P-selectin is released by activated endothelial cells and platelets; prior reports describe sP-selectin levels as being both elevated [23] and decreased [24] in CLD, which suggests that circulating levels of sP-selectin are likely context-dependent. Recently, however, high sP-selectin levels were shown to have a positive predictive value of the presence of PVT following splenectomy secondary to portal hypertension [25]. Alternatively spliced tissue factor (asTF), the soluble isoform of tissue factor initially described in 2003 [26], may also be released by activated endothelial cells [27] and has been recently shown to be elevated in CLD [28]. Unlike the highly coagulant full-length form of tissue factor, circulating asTF protein is only minimally coagulant and therefore unlikely to be consumed in clotting reactions, which renders it a potentially attractive biomarker.

In 2021, Turon et al. reported the results of the largest prospective study to date that explored the possibility of using clinical, ultrasonographic, and/or hemostatic factors to predict PVT in CLD patients [29]. The authors reported that the factors predictive of PVT were mostly those related to the severity of portal hypertension, and not hemostatic alterations and/or inflammatory markers. The finding regarding the importance of portal hypertension is in agreement with findings reported by other groups [30, 31]. However, Turon et al. also observed that activity levels of several hemostatic proteins, including FV, FVIII, PC, and PS, were significantly lower at enrollment in those patients who developed PVT whereas sP-selectin levels were higher; unfortunately, no information was available as to whether these parameters track with disease severity in this “CTP-A heavy” cohort [29]. Hence, we carried out an exploratory prospective study to evaluate a possible relationship between CLD severity and coagulation factor activity levels.

## Methods

### Study subjects and sample collection

In Cohort I, whole blood was collected via venipuncture (0.129 mol/L sodium citrate) from healthy subjects (n = 30) and CLD patients suffering from cirrhosis due to untreated hepatitis C infection (HCV) and/or excessive alcohol consumption seen at an outpatient clinic at Hôpital Foch (Suresnes, France) that were previously assigned a CTP score: CTP-A, n = 12; CTP-B, n = 19; CTP-C, n = 11. All subjects were recruited within 1 year; there were no diagnosed cases of PVT in this cohort, which precluded the need for follow-ups related to this condition. Within 2 h post-collection, specimens were centrifuged for 15 min at 1500×*g*, and then 13,000×*g* for 2 min to obtain platelet poor plasma (PPP). A sample of the PPP was immediately assessed for FV activity using Stago STA-Deficient V assay kit and the remainder of the PPP was frozen at − 80 °C until used.

In Cohort II, blood was collected via venipuncture (0.129 mol/L sodium citrate) from CLD patients awaiting liver transplant at University of Cincinnati. 43 patients were recruited over the course of 1 year. 33 patients received LT within a year following enrollment. Of those who received LT, 28 were still alive at 1-year follow-up. 5/10 patients who did not receive LT, were living at 1-year follow-up. CTP and MELD-Na scores were assessed at the time of blood collection. Cohort II comprised patients suffering from alcoholic and non-alcoholic steatohepatitis, with 9/43 experiencing an HCV comorbidity. The cohort consisted of CTP-A, n = 12; CTP-B, n = 28; CTP-C, n = 3; all subjects gave informed consent. Within 2 h post-collection, specimens were centrifuged for 15 min at 1500×*g*, and then 13,000×*g* for 2 min to obtain PPP. A sample of the PPP was immediately assessed for FV activity as in Cohort I. Patient demographics and plasma-based assay results are presented in Table 2; 6-month outcomes were determined by chart review. For the majority of subjects in Cohort II, CT/MRI/Doppler ultrasound imaging studies were carried out; a small minority of the subjects did not have radiology follow up because they received the transplant shortly after being enrolled in the study.

For both cohorts, the following inclusion and exclusion criteria were applied. Anyone 18 years of age or older with clinical, radiological, or biochemical evidence of liver cirrhosis; irrespective of gender, ethnicity or race was considered eligible to participate in this study. The following subjects were excluded: anyone under the age of 18, adults unable to consent, prisoners, pregnant women (women were assumed to be pregnant if they exhibited any of the presumptive signs of pregnancy, such as a missed menses, until the results of a pregnancy test were negative), women with recent (within last 3 months) use of oral contraceptives. The rationale for excluding pregnant women and those with recent use of oral contraceptives is the hormonal changes that can increase the risk of thromboembolism. Individuals with inherited hypercoagulable states such as FV Leiden, prothrombin gene mutation, PC deficiency, PS deficiency, antithrombin deficiency, increased FVIII levels were also excluded. Individuals with myeloproliferative disorders, abdominal inflammatory lesions including infection, pancreatitis, and inflammatory bowel disease were excluded because all these conditions alter the risk of PVT.

### Plasma based assays

PPP was obtained by centrifugation of whole blood anticoagulated with sodium citrate and utilized for factor quantification using factor-deficient STA assays (Diagnostica Stago) to measure the activity levels of FV, FVIII, PC, PS, and thrombomodulin (TM). D-dimer levels were measured using STA-liatest D-Di (Diagnostica Stago). The levels of sP-selectin protein were measured in PPP using commercially available ELISA (Millipore Sigma RAB0426). The levels of asTF protein were measured in PPP using custom ELISA as previously described [32].

### Statistical analysis

**Fig. 1:**
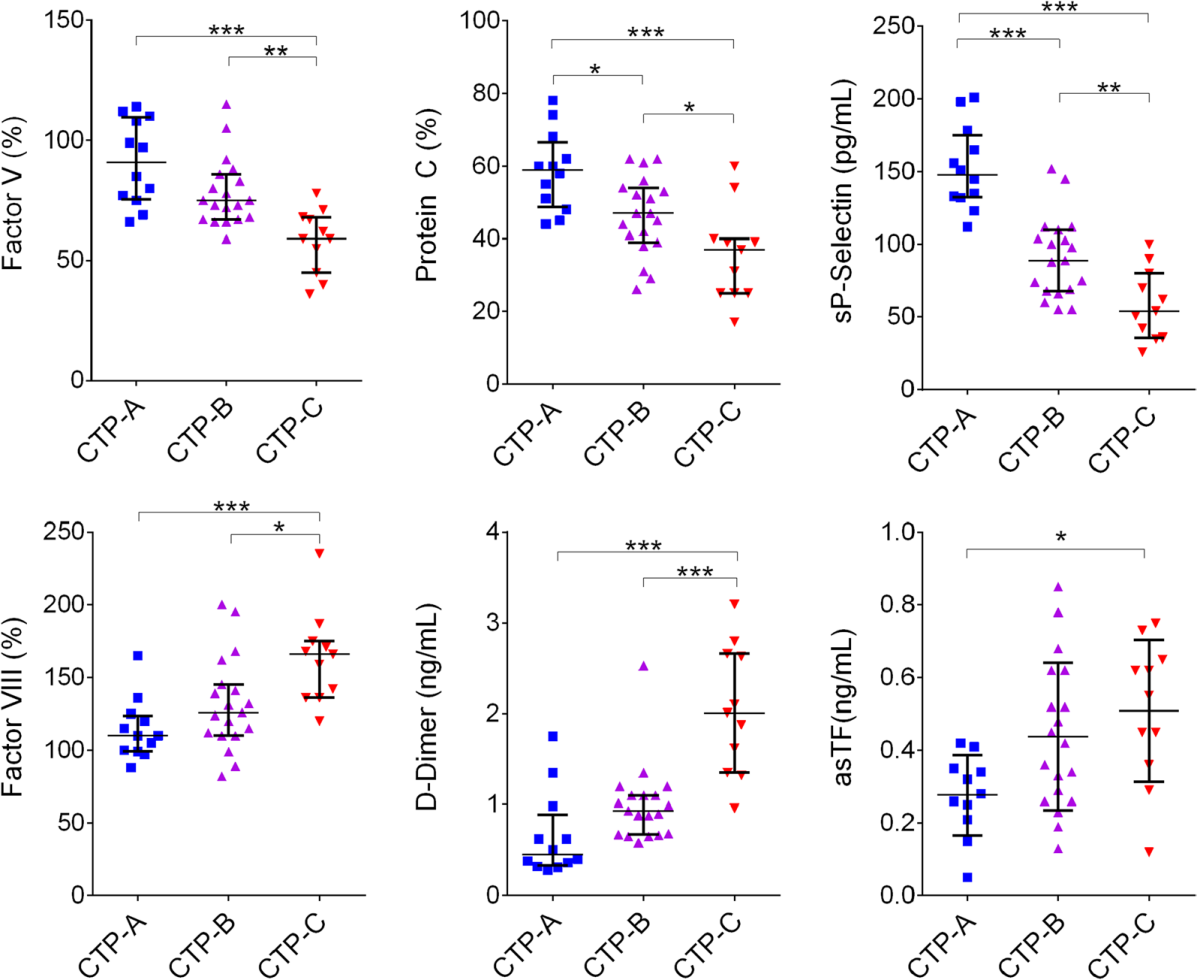
Factor activity (%) or concentration levels in PPP collected from ambulatory CLD subjects; CLD severity was scored using the Child–Turcotte–Pugh scoring system (CTP-A, CTP-B, or CTP-C). (*p < 0.05, **p < 0.01, ***p < 0.001)

**Fig. 2:**
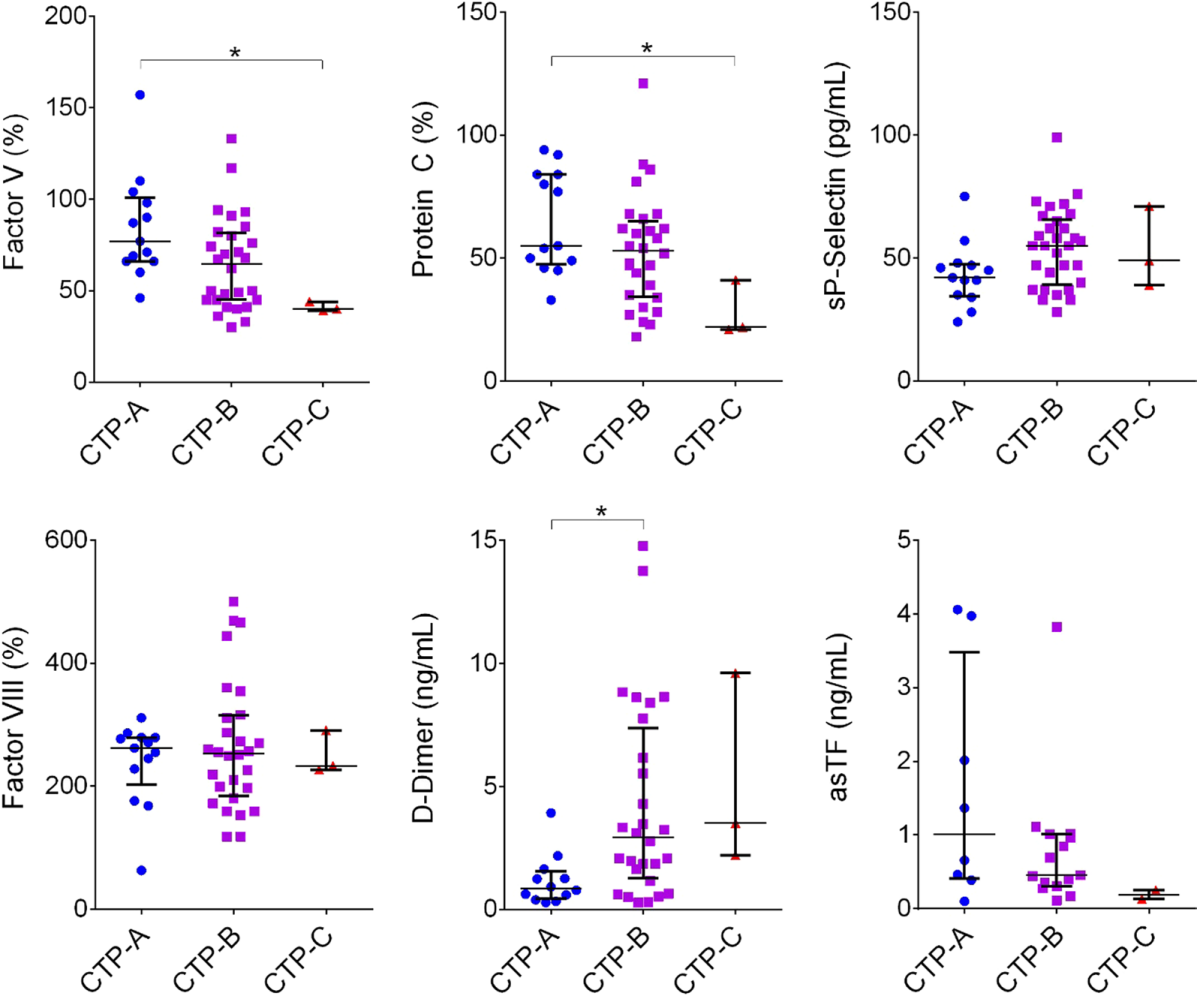
Factor activity (%) or concentration levels in PPP collected from CLD patients awaiting liver transplant; CLD severity was scored using the Child–Turcotte–Pugh scoring system (CTP-A, CTP-B, or CTP-C). (*p < 0.05)

One-way ANOVA analysis was performed using GraphPad Prism (version 6.0; GraphPad Software, San Diego, CA) and used to detect the differences between groups in Figs. 1 and 2, with *p* of 0.05 being considered significant. Linear regressions between factor activity levels or plasma concentrations and MELD-Na were performed using GraphPad Prism 6.0.

Multiple linear regression with MELD-Na − 3.78 × ln(bilirubin) − 9.57 × ln(creatinine) (coefficients determined by Kamath et al. [33]) as dependent variable and ln(FV%) and ln(PC%) as independent variables were used to derive the coefficients for ln(FV%) and ln(PC%) along with the intercept yielding our novel CLD scoring formula. All analyses were performed using SAS Version 9.4 (SAS Institute, Cary, NC). Two logistic regression analyses were performed to develop the compensation score for the prediction of PVT. For the first logistic regression analysis, PVT incidence was dependent, ln(FV%) and ln(FVIII%) were independent variables; for the second logistic regression analysis, PVT incidence was dependent, ln(PS%) and ln(FVIII%) were independent variables. Their coefficients were used to create the two compensation scores.

## Results

### Cohort I: ambulatory patients

We first sought to determine whether changes in the activity levels of select coagulation factors track with CLD severity in a single ambulatory cohort in which no instances of PVT were identified. PPP specimens of CLD patients seen at an outpatient ambulatory clinic were first assessed for the activity levels of FV, FVIII, PC, PS, and thrombomodulin (TM) along with the circulating levels of sP-selectin and asTF. As expected, FV activity progressively decreased along the spectrum of CLD severity (Fig. 1). PC and PS activity levels also decreased with CLD severity (Table 1). FVIII and TM activity progressively increased with disease severity (Fig. 1, Table 1). The circulating levels of D-dimer along with those of asTF protein increased with disease severity, while the levels of sP-selectin decreased (Fig. 1).

**Table 1:** Factor activity (%) and protein concentration levels in PPP collected from ambulatory CLD patients previously scored as A, B, or C using the Child-Turcotte-Pugh scoring system (CTP-A, CTP-B, or CTP-C)

	Healthy controls (n = 30)	CTP-A (n = 12)	CTP-B (n = 19)	CTP-C (n = 11)	*p* value
FV(%)	101 [92–116]	91 [77–109]	74 [68–85]	59 [50–68]	< 0.0001
FVIII (%)	98 [87–124]	110 [100–121]	125 [111–143]	166 [139–173]	< 0.0001
PC (%)	132 [121–135]	59 [50–64]	47 [40–54]	37 [25–40]	< 0.0001
PS (%)	79 [76–86]	56 [47–60]	46 [38–52]	42 [38–51]	< 0.0001
TMa (%)	100 [92–106]	137 [120–198]	142 [128–164]	158 [152–226]	< 0.0001
D-Di(ng/mL]	0.23 [0.22–0.32]	0.45 [0.35–0.71]	0.92 [0.68–1.10]	2.01 [1.49–2.65]	< 0.0001
sP selectin (pg/m]	136 [119–154]	148 [133–168]	89 [69–107]	54 [39–75]	< 0.0001
asTF(ng/mL)	0.23 [0.21–0.26]	0.30 [0.24–0.37]	0.39 [0.28–0.27]	0.55 [0.41–0.64]	< 0.0001

Presented as median with interquartile range; *p* values were assessed via one-way ANOVA to determine if the variance across the groups was significant

### Cohort II: LT candidates

We next sought to evaluate more severe CLD, i.e., patients with advanced disease awaiting LT. 43 (mostly CTP-B) LT candidates were enrolled in this study which included access to disease history, severity scoring, standard lab results, and limited outcomes. PPP specimens were evaluated for the activity levels of FV, FVIII, PC, PS, and the levels of D-dimer, sP-selectin, and asTF along with standard CLD evaluation labs: serum sodium, albumin, creatinine, total bilirubin, and PT/INR (Table 2). As in Cohort I, FV and PC activity declined significantly with CLD progression, while the levels of D-dimer rose (Fig. 2). Also in agreement with the Cohort I were the low levels of PS activity in all patients (Table 2). FVIII activity was markedly elevated across the entire Cohort II, while sP-selectin protein levels were universally low (Fig. 2). In agreement with our previous study, the levels of asTF protein in the majority of subjects were higher than those we reported in healthy individuals [28] (Fig. 2).

**Table 2:** Demographics and clinical characteristics of CLD patients awaiting liver transplant scored as A, B, or C using the Child-Turcotte-Pugh scoring system (CTP-A, CTP-B, or CTP-C)

	CTP-A (n = 12)	CTP-B (n = 28)	CTP-C* (n = 3)	*p* value
Age	59.5 (56.5–61.5)	59 (52–65)	55 (41–69)	0.9678
Sex [n (%)]				
Male	11 (92)	18 (64)	3 (100)	
Female	1 (8)	10 (36)	0 (0)	
Race (n)				
White	11	27	3	
Black	0	1	0	
Other	1	0	0	
Etiology (n)				
ASH	7	10	2	
NASH	7	16	1	
HCV	6	2	1	
Mixed liver disease	8	3	1	
Portal vein thrombosis	1	2	1	
MELD-Na score	10 (9–12)	15 (11–17)	19 (16–20)	0.0022
Serum sodium	139 (138–140)	137 (135–138)	138 (134–139)	0.0281
Serum creatinine	0.79 (0.73–1.26)	0.91 (0.71–1.23)	1.02 (0.79–1.12)	0.9540
Total bilirubin	1.1 (0.7–1.4)	1.9 (1.3–2.7)	5.2 (2.4–6.9)	0.0009
PT/INR	1.2 (1.1–1.3)	1.4 (1.2–1.5)	1.6 (1.5–1.7)	0.0019
Serum albumin	3.8 (3.7–3.9)	3.4 (3.2–3.7)	2.5 (2.5–3.2)	0.0019
FV (%)	79 (66–100)	65 (45–81)	40 (39–44)	0.0175
FVIII (%)	259 (215–278)	253 (193–312)	233 (227–291)	0.6888
PC (%)	66 (48–84)	53 (35–63)	22 (21–41)	0.0346
PS (%)	59 (47–76)	59 (50–67)	66 (61–69)	0.5805
D-Di (ng/m)	0.85 (0.54–1.36)	2.94 (1.52–6.56)	3.50 (2.20–9.61)	0.0345
sP selectin (pg/m)	43 (39–47)	57 (41–67)	49 (39–71)	0.0973
asTF (ng/m)	1.011 (0.445–2.503)	0.456 (0.333–0.986)	0.133, 0.254**	0.1725
Novel CLD score	11.2 (9.4–12.8)	14.8 (12.5–18.3)	21.8 (21.3–22.5)	0.0032

Presented as median with interquartile range. *median, min, and max reported due to low n. **only 2 CTP-C patients had detectable levels of asTF, both values given. p values were assessed via one-way ANOVA to determine if the variance across the groups was significant

### Factor V and protein C activity levels can replace PT/INR in MELD scoring

**Fig. 3:**
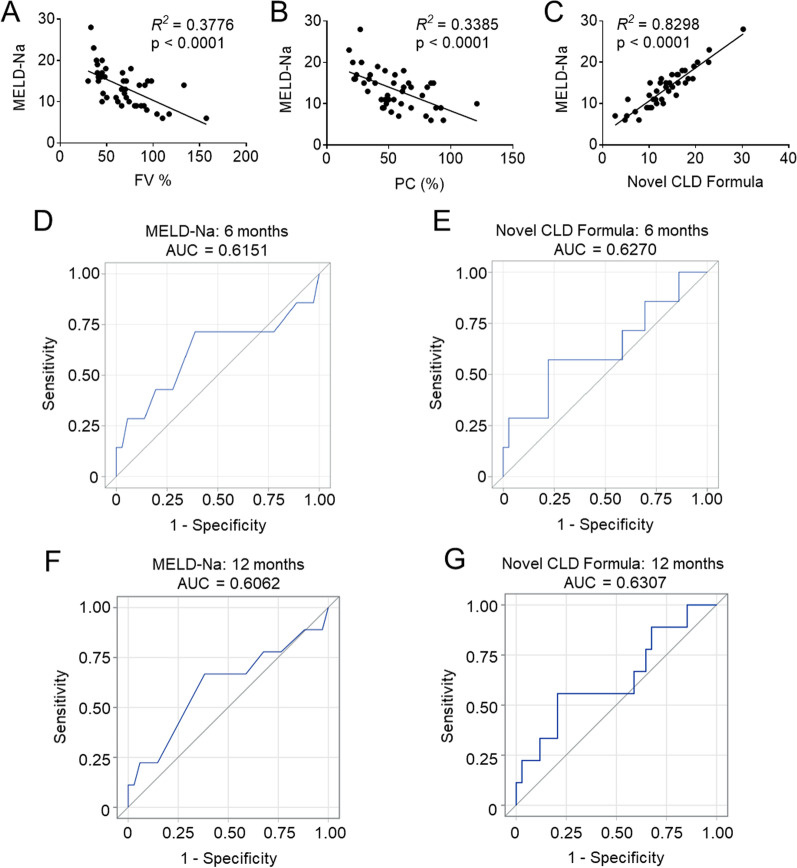
Correlations of factor activity levels and novel CLD scoring system with MELD-Na and PT/INR. **A** Correlation of FV activity levels with MELD-Na. **B** Correlation of PC activity levels with MELD-Na. **C** Correlation of novel CLD scoring system with MELD-Na. Receiver operating characteristic curves for 6-month mortality for **D** MELD-Na and **E** novel CLD formula. Receiver operating characteristic curves for 1-year mortality for **F** MELD-Na and **G** novel CLD formula

We investigated whether factor activity levels could be used to calculate a CLD severity score for LT candidates. The current gold standard for tracking CLD progression in LT candidates, MELD-Na, assigns a score based on a patient’s levels of bilirubin and creatinine (indicators of liver and kidney metabolic health, respectively) as well as PT/INR. Monitoring coagulation is necessary as the liver is the main site of synthesis for pro- and anti-hemostatic factors. Expression of these factors is increasingly deranged with worsening disease severity. Given that PT/INR only measures clot formation and not dissolution, the appropriateness of its use to assess coagulative potential in CLD patients is debatable; we hypothesized that measuring activity levels of key hemostatic factors may serve as a non-inferior option to the use of PT/INR in MELD-Na scoring. Correlation analyses of factor activity levels in LT subjects and their MELD-Na scores revealed that FV activity levels significantly correlated with MELD-Na values; interestingly, activity levels of anti-coagulant Protein C behaved analogously to those of FV levels (Fig. 3A, B). Seeing that activity levels of both a pro-coagulant (FV) and an anti-coagulant (PC) protein strongly correlated with MELD, we next sought to mathematically combine the FV and PC activity levels. Thus, we performed multiple linear regression using the constants for bilirubin and creatinine established by Kamath et al. [33] as the dependent variable and the natural logs of FV and PC activity as independent variables. This multivariate analysis enabled the creation of a novel scoring formula:

$$\begin{aligned} {\text{CLD Score }} & = { 3}.{78 } \times {\text{ ln}}\left( {bilirubin} \right) \, + { 9}.{57 } \times {\text{ ln}}\left( {creatinine} \right) \, {-}{ 1}.{16 } \times {\text{ ln}}\left( {FV\% } \right) \\ & \quad {-}{ 3}.{1}0 \, \times \;{\text{ln}}\left( {PC\% } \right) \, + { 29} \\ \end{aligned}$$

Assessment of our novel formula vs MELD-Na showed a strongly significant positive correlation (Fig. 3C). At the time of 6-month follow-up, 36 of the 43 LT patients were alive. The seven deceased patients had an average MELD-Na of 15.2 ± 6.4 and an average novel CLD score of 16.2 ± 6.9. Using the c-statistic with 6-month mortality as the endpoint, the area under the receiver operating characteristic (ROC) curves for MELD-Na and our novel scoring formula did not differ significantly. The areas under the curves were 0.615 and 0.627, respectively (Fig. 3D, E). At the time of 1-year follow-up, 33 of the 43 LT patients were living. Using c-statistic with 12-month mortality as the endpoint, the AUC under the ROC were 0.606 and 0.631 for MELD-Na and our novel scoring formula, respectively (Fig. 3F, G). These findings suggest that our novel formula is likely non-inferior to MELD-Na at predicting 6-month and 1-year mortality.

### PVT is associated with low factor VIII, factor V, and protein S activity levels

**Fig. 4:**
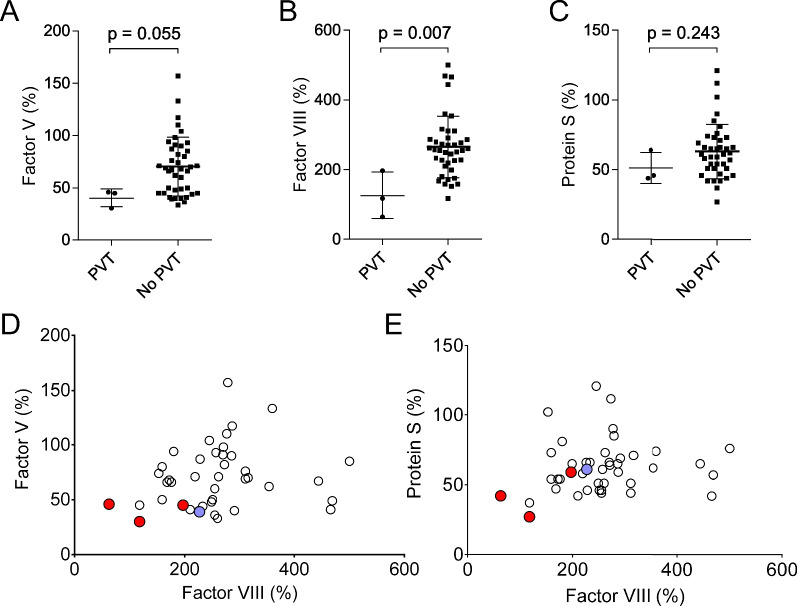
**A**–**C** Activity levels of FVIII, FV, and PS in patients with CLD awaiting LT. **D**, **E** FV and PS activity levels with patient-matched FVIII activity levels. Red dots: patients with confirmed PVT within 3 months of enrollment; blue dot: patient with confirmed PVT 25 months prior to enrollment

PVT is a major complication of CLD, and our ability to effectively detect and/or predict it is lacking. In our cohort of 43 LT patients, there were 3 instances of PVT diagnosed by imaging within 3 months of consent (7%). Patients with diagnosed PVT were followed up clinically via several visits to our LT clinic post-diagnosis; CBC counts were recorded and there were also biochemical and imaging follow-ups comprising updated MELD and CT/MRI/Doppler ultrasound, respectively. Post-hoc correlation analysis between PVT incidence and the measured parameters revealed a strongly significant inverse correlation with FVIII activity levels (*p* = 0.010; Table 3). Activity levels of two other factors, FV and PS, also displayed in-trend inverse correlations with PVT incidence (*p* = 0.069 and *p* = 0.064, respectively; Table 3). Next, we performed Mann–Whitney tests to compare the activity levels of FVIII, FV, and PS for patients with and without PVT (Fig. 4A–C). Again, FVIII levels were found to be significantly lower in patients with PVT, while FV levels remained in-trend; however, PS levels, while lower in patients with PVT, were not statistically significantly different between the groups. Given the strength of the correlation between PVT incidence and FVIII activity, we proceeded to match and graph each patient’s FV activity levels with the corresponding FVIII activity levels; we also graphed FVIII levels vs PS levels, given the in-trend value obtained for PS in our correlation analysis (Figs. 4D, E). When distances from the graph origin were calculated for FV:FVIII, the three patients with PVT were 1st, 2nd, and 10th closest to the origin (Fig. 4D); in the graph of PS:FVIII they were 1st, 2nd, and 11th closest to the origin (Fig. 4E). We carried out logistic regression analysis to use the activity levels of FVIII, along with those of FV and PS, to create two compensation score equations, CS-1 and CS-2. The equations are as follows:

$${\text{CS}} - {1 } = \, - {6}.0{9 } \times {\text{ ln}}\left( {FV\% } \right) \, {-}{ 4}.{66 } \times {\text{ ln}}\left( {FVIII\% } \right) \, + { 48}$$

$${\text{CS}} - {2 } = \, - {2}.{27 } \times {\text{ ln}}\left( {PS\% } \right) \, {-}{ 4}.{34 } \times {\text{ ln}}\left( {FVIII\% } \right) \, + { 5}0.{5}$$

**Table 3:** Correlation analysis, incidence of PVT versus indicated parameters

Parameter	Pearson correlation coefficient	(r)
Age	− 0.094	0.550
Gender	− 0.049	0.757
Plasma FV activity	− 0.280	0.069
Plasma FVIII activity	− 0.386	0.010
D-Dimer	0.225	0.146
Plasma PC activity	− 0.096	0.541
Plasma PS activity	− 0.285	0.064
sP selectine	− 0.205	0.199
Serum sodium	0.058	0.713
Serum creatinine	− 0.228	0.142
Serum albumin	− 0.063	0.687
Total bilirubin	− 0.008	0.962
INR	0.188	0.233
Hepatic encephalopathy	− 0.098	0.533
Jaundice	− 0.099	0.526
Ascites	0.099	0.526
Variceal bleeding	− 0.141	0.367
CTP score	0.083	0.598
MELD-Na	0.070	0.657
*Comorbidities*		
Excessive alcohol consumption	0.124	0.428
Hepatitis C	0.308	0.045
HCC	0.104	0.508

A total of 11 patients, including the 3 cases of confirmed PVT, had a CS-1 score > 0 while a total of 20 patients had a CS-2 score > 0. None of the patients with a CS-1 or CS-2 less than 0 was diagnosed with PVT. 2 of the 3 patients with a confirmed PVT had CS-1 > 5.00 and CS-2 > 8.00. One of them, who underwent LT one day after sample collection, was found to have PVT at the time of transplant. This patient was not on anticoagulants at the time of sample collection and had a CS-1 of 5.06, a CS-2 of 8.20 (FV 30%; FVIII 118%; CTP-B; MELD-Na 15). The other patient was diagnosed with PVT by contrast CT of the abdomen 3 months prior to enrollment; at the time of enrollment and sample collection, the patient was not on anticoagulants and had a CS-1 of 5.38, CS-2 of 8.43 (FV 46%; FVIII 63%; CTP-A; MELD-Na 12), The third patient with a confirmed PVT was diagnosed 10 days after enrollment via contrast CT of the abdomen. The patient was not on anticoagulants at the time of sample collection, had a CS-1 of 0.19, a CS-2 of 1.05 (FV% 45; FVIII 197%; CTP-B; MELD-Na 17). Multi-factor consumption may thus be a possible unifying condition among the three patients with PVT.

## Discussion

Here we report that a panel of routinely performed point-of-care plasma assays display the potential to aid in CLD management. In this exploratory study, we demonstrate that the activity levels of FV and PC may hold utility as an alternative means of tracking the hemostatic status in LT candidates. In addition, we have charted an approach to devising a scoring system that may have utility to assess short(er)-term risk for PVT in LT. While our study is limited by its small cohort sizes, the parameters we chose to examine allowed robust correlation analyses to identify coagulation factors whose activity levels correlate not only with the presence of PVT, but also with the severity of CLD. Large, multi-center studies are required to further evaluate the feasibility of using FV and PC activity levels as a substitute for PT/INR in MELD scoring, especially with regard to the clinical outcomes, and to corroborate our approach to assess the risk for PVT.

Since its introduction in 2002, MELD has helped reduce the mortality rate for those on the LT waiting list by 12% [34]. It is far superior at predicting short-term mortality than CTP scoring [35]. However, MELD is not perfect, and its modification may further improve outcomes. Prior to 2016, hyponatremia was a risk factor for waitlist mortality. This was solved by incorporation of serum sodium levels in MELD scoring (MELD-Na) [36]. However, MELD-Na still has problematic features e.g. the use of PT/INR, which is a poor indicator of hemostatic balance, and the use of creatinine levels, which are directly related to a patient’s muscle mass, underrepresents the severity of disease in women and those with poor nutritional status [37].

We found that even in a relatively small LT cohort, substituting PT/INR with the activity levels of FV and PC provided a non-inferior method to predict 6-month and 1-year mortality compared to MELD-Na. This is likely attributable to the central nature of these two proteins in the coagulation cascade and how their circulating levels are reflective of hemostatic rebalancing in CLD. Activated FV (FVa) is an essential component of prothrombinase, and active PC regulates FVa by proteolytic cleavage; as such, FV and PC activity levels comprise key indicators of hemostatic capacity. As shown in Figs. 1 and 2, deficiency in these factors strongly correlates with CLD severity in the ambulatory cohort, and in the LT cohort. While maintaining the speed of access to clinical laboratory results, FV and PC activity quantification gains advantage over PT/INR due to the greater dynamic range of factor quantification measurements over clotting time measurements, which are typically accurate to only a tenth of a second. Monitoring the activity of both a coagulant and an anti-coagulant plasma factor is likely qualitatively superior to measuring the duration of an artificially induced blood clotting process. Larger cohort sizes are certainly needed to validate these findings and determine the full benefit of using FV and PC activity levels for predicting outcomes.

PVT is a serious complication present in ~ 5–25% of CLD patients. Consistent with these previous reports, PVT were identified in 3/42 LT patients (7.1%). Occlusive PVT at the time of LT is associated with an up to seven times increased risk of death 30 days post-op [2–4]. The fact that enoxaparin both prevents PVT formation in early CLD and slows disease progression indicates that these pathophysiologic processes are likely linked [38]. It was reported that PVT could be predicted based on CLD etiology, MELD score, ascites severity, and race [39]; however, this method only minimally takes into account a patient’s hemostatic state. Recently, Turon et al. reported that portal hypertension, portal blood flow velocity, and a history of variceal bleeding comprise the primary risk factors for development of PVT in cirrhotic patients [29]. Turon et al. also reported that measuring hemostatic factor activity does not seem to predict the development of PVT. The seeming discrepancy of our findings with that of Turon et al. is likely due to the differences in study design. The Turon study prospectively enrolled patients with cirrhosis and followed the patients long-term (an average of 4 years). However, hemostatic parameters were only measured at the time of consent. Given that 72% of the patients in that study were CTP-A, these patients were likely not yet in a de-compensated state. In contrast, our study assessed patients who had experienced PVT within a narrowly defined time interval, i.e. within only 3 months of consent/blood collection.

In patients awaiting LT, monitoring the activity of FVIII along with either FV and/or PS may comprise a straightforward, “point-of-care friendly” means to assess short-term risk for PVT. We observed that in patients with severe CLD who had FV activity below 50% and FVIII activity below 200%, which corresponded to a CS ≥ 0, the incidence of PVT rose drastically. In fact, of the 6 patients who had FV and FVIII activity levels less than 50% and 200%, respectively, 3 had a confirmed PVT. Thus, FV and FVIII activity below these thresholds may very well comprise a harbinger of hemostatic decompensation and/or cofactor consumption in CLD. As a consequence of hemostatic decompensation and increased production of VWF, FVIII activity levels are progressively elevated with worsening CLD severity. However, similar to the findings of Fimognari et al. [13], we found lower levels of plasma FVIII activity to correspond to PVT occurrence. Our cohort size is small and the observed incidence of PVT low; nonetheless, when our data is viewed in the context of Fimognari et al. [13], it raises the possibility that above-normal FVIII activity levels are necessary for hemostatic rebalancing in patients with CLD. Whether declining FVIII activity levels are the cause of, or a result, of consumptive coagulopathy remains to be elucidated. However, it seems likely that plasma activity levels of FVIII, which would be in the “normal range” for a healthy individual, should be viewed with concern in the CLD patient, especially in more severe cases. Longitudinal multi-center studies are necessary to corroborate our findings regarding the potential utility of FV and/or PS activity levels, in combination with those of FVIII, to assess the risk of PVT in CLD.

The constants used in MELD-Na were based on correlations with short- and long-term mortality rates in four large cohorts awaiting LT [33]. Our study is limited by its modest cohort sizes, relatively low incidence of PVT, and short-term patient outcomes (6 months and 1 year). Nonetheless, we posit that the novelty of our findings mitigates these limitations: we have mapped a new path forward to improve the hemostatic-assessment component of MELD-Na and, possibly, evaluate the propensity for PVT among LT candidates.

## Conclusion

We demonstrate for the first time that plasma activity levels of FV and PC might have the potential to substitute PT/INR in MELD scoring. In addition, we show the potential of using the combination of FV and FVIII plasma activity levels to assess the risk of PVT in CLD. Longitudinal multi-center studies featuring larger cohorts are needed to corroborate our findings.

## Data Availability

Requests should be sent to the corresponding author, Dr. Vladimir Y. Bogdanov (vladimir.bogdanov@uc.edu).

## Abbreviations

asTF: Alternatively spliced tissue factor
CLD: Chronic liver disease
CS: Compensation score
CTP: Child–Turcotte–Pugh
ETP: Endogenous thrombin potential
FV: Factor V
FV:PC: Factor V and protein C activity composite score
FVIII: Factor VIII
HCV: Hepatitis C infection
MELD: Model for end-stage liver disease
LT: Liver transplant
MELD-Na: Model for end-stage liver disease with sodium
PPP: Platelet poor plasma
PVT: Portal vein thrombosis
PC: Protein C
PS: Protein S
PT/INR: Prothrombin time/international normalized ratio
vWF: von Willebrand factor
